# Game Experience and Learning Effects of a Scoring-Based Mechanic for Logistical Aspects of Pediatric Emergency Medicine: Development and Feasibility Study

**DOI:** 10.2196/21988

**Published:** 2021-03-11

**Authors:** Cevin Zhang, Jannicke Baalsrud Hauge, Karin Pukk Härenstam, Sebastiaan Meijer

**Affiliations:** 1 School of Media and Design Beijing Technology and Business University Beijing China; 2 Department of Biomedical Engineering and Health Systems Kungliga Tekniska Högskolan Huddinge Sweden; 3 Department of Sustainable Production Development Kungliga Tekniska Högskolan Södertalje Sweden; 4 Pediatric Emergency Department Karolinska University Hospital Stockholm Sweden

**Keywords:** serious game, emergency department, experience, learning engagement, learning behavior transition, logistical performance

## Abstract

**Background:**

Using serious games for learning in operations management is well established. However, especially for logistics skills in health care operations, there is little work on the design of game mechanics for learning engagement and the achievement of the desired learning goals.

**Objective:**

This contribution presents a serious game design representing patient flow characteristics, systemic resource configurations, and the roles of the players based on a real Swedish emergency ward. The game was tested in a set of game-based learning practices in the modalities of a physical board game and an online multiplayer serious game that implemented the same game structure.

**Methods:**

First, survey scores were collected using the Game Experience Questionnaire Core and Social Presence Modules to evaluate the experience and acceptance of the proposed design to gamify real processes in emergency care. Second, lag sequential analysis was applied to analyze the impact of the game mechanics on learning behavior transitions. Lastly, regression analysis was used to understand whether learning engagement attributes could potentially serve as significant predicting variables for logistical performance in a simulated learning environment.

**Results:**

A total of 36 students from courses in engineering and management at KTH Royal Institute of Technology participated in both game-based learning practices during the autumn and spring semesters of 2019 and 2020. For the Core Module, significant differences were found for the scores for negative affect and tension compared with the rest of the module. For the Social Presence Module, significant differences were found in the scores for the psychological involvement – negative feelings dimension compared with the rest of the module. During the process of content generation, the participant had access to circulating management resources and could edit profiles. The standard regression analysis output yielded a ΔR^2^ of 0.796 (F1_4,31_=2725.49, *P*<.001) for the board version and 0.702 (F2_4,31_=2635.31, *P*<.001) for the multiplayer online version after the learning engagement attributes.

**Conclusions:**

The high scores of positive affect and immersion compared to the low scores of negative feelings demonstrated the motivating and cognitive involvement impact of the game. The proposed game mechanics have visible effects on significant correlation parameters between the majority of scoring features and changes in learning engagement attributes. Therefore, we conclude that for enhancing learning in logistical aspects of health care, serious games that are steered by well-designed scoring mechanisms can be used.

## Introduction

Emergency departments (EDs) are responders in the immediate aftermath of incidents and catastrophic situations and the foremost part of the health care system that delivers care around the clock. EDs are faced with the challenge to create a sustainable working environment despite rapidly shifting demands and preconditions for work [1]. Resilient operations are often associated with well-performing dynamic resource management and adaptive coordination to facilitate sustainable, safe work practices.

The ability to manage resources, especially when human resources are key in health care organizations, is a nontechnical skill (NTS) that requires decision making, coordination, and leadership. NTSs are defined as cognitive, social, and personal skills related to organizational robustness and resilience in management [2]. Coordination, decision making, and situational awareness are cornerstone NTSs in, amongst others, air transport [3], emergency medical services provision [4], anesthetist training [5], and nursing situations [6] in the request of maximum levels of operational safety and quality under conditions of stress and disruption. Practices such as staffing strategies and controlling, rostering, and capacity building in human resource management are important organizational strategies for creating preconditions for resilient practices for the operators who encounter the most problems [7].

Technology-enhanced learning, as a broad category of applying technology in medical and health service training situations, can be used to teach NTSs related to resource management. There are proven benefits of using a learning environment in which the learners play an active role and rely on modeling real systems, experiential learning, debriefing, evaluating play, and systematic analysis [8]. Such learning environments may be simulations and games that are used to train individuals and teams until they are technically proficient in the skills needed for a collective, reliable, high-performance working system [9,10]. The usage of serious games for educational purposes has a long history within military and engineering education [11,12]. Within medical and health care education, serious games are used for procedural training in surgery [13], anesthesia [14], and obstetrical clinical practice [15] and to enable managers to improve teamwork coordination [16], communications [17], and decision making [18,19]. A good overview of how games, specifically simulation-based games, are used for medical education can be found in Wang et al [10].

A longstanding challenge is giving the players feedback while they play in such a way that supports the learning process, specifically if the topic has a complex nature. The common method is to have experienced facilitators observe the gameplay (ie, external feedback), and often learning takes place during the debriefing session. Inbuilt mechanics like points and, in some cases, also badges and leaderboards are the most commonly used game mechanics [20] but are often disconnected from the learning experience. A different mechanic is used in math games for kids, where there is a simple right and wrong answer and the mechanics put in place are corrective and encourage retries. However, to strengthen the learning in more complex environments, stealth assessment has been more and more embedded into serious games. Progress in the field of serious games necessitates that we evaluate how game mechanics can impact learning engagement. Learning performance improvements can be expected to be the achievement goals of game-based training activities after the players have become situationally motivated through gamified processes. Recently, researchers have advanced this field from pure learning to performance-forecasting studies by taking advantage of learning analytics methods [21-23], learning benefits diagnoses, and analytical modeling based on learning behavior trajectories. The pursuit of a challenging situation and willingness to learn from the consequences of decision making are the prevalent motivation drivers in virtual environments [24]. For a contextually appropriate training and educational game design, motivational and flow experience theories provide excellent guidelines for mapping psychological needs and gaming mechanics selections [25,26].

Although recent health care simulations have been applied to identifying learners’ gameplay processes [27]; understanding their knowledge acquisition process, motivation, or behavior [28]; and assessing their learning performance based on a virtual reality representation of medical complexities [29], there is a knowledge gap regarding how logistical aspects of health care operations can be gamified to encourage positive changes in learning engagement when training and practicing nontechnical decision-making skills related to essential logistical features [30]. Whether scoring-based game mechanics based on the logistical features of the health care system can be efficiently used as attributes of learning engagement has yet to be explored. Serious game analytics may open opportunities for a better understanding of engagement in game-based learning environments [31]. In addition, there are no human-computer simulation practices in recently published gaming articles on the delivery of care in health care organizational settings nor are there scoring systems to gamify the logistics of care within a pediatric ED [32], even though the scoring system is the most frequently used gamification element in comparison with badges, leaderboards, avatars, levels, and rewards.

Based on these observations, the following research question is asked in this study: Does a scoring-based game mechanic closely connected to the patient flow and logistical processes of an emergency department motivate players to interact with artifacts in a simulated emergency care unit?

The ability to motivationally interact with learning artifacts is recognized as engagement in a learning process [33]. The conceptual link between learning and engagement refers to the adoption of cognitive strategies based on self-regulation of performance in learning processes, with both theoretical models converging on the belief that more efficient and higher-quality engagement is available when students use the necessary cognitive knowledge and skills. The learning engagement attributes should accentuate the order of magnitude at which the players are influenced by gamification features. It is also interesting to explore whether the attributes themselves can explain logistical performance in a health care production serious game. Since the object of this study is to recognize how students experienced the use of the serious game and to explore the extent to which the proposed scoring-based mechanic influences learning engagement, the overall research question in our study is operationalized in the following research subquestions: (1) In what ways does the use of the logistical serious game influence students’ experience? (2) How, if at all, does the use of the scoring-based mechanic enrich learning engagements?

Based on previous studies’ usage of the number of initiated tasks and finished tasks as learning engagement attributes [31], we addressed relationships between logistical performance and the following traits, for which a regression model was performed:

- Is the number of initiated activities for resource content editions positively related to logistical performance in the serious game?
- Is the number of initiated activities for patient profile updates and investigations positively related to logistical performance in the serious game?
- Is the number of invitations for cooperation positively related to logistical performance in the serious game?
- Is the number of finished activities positively related to logistical performance in the serious game?

To answer these research questions, this study designed a logistical outcome-based scoring system and evaluated its effectiveness and acceptance in a fully pledged game-based training and learning exercise.

## Methods

### Game Design

This section presents the design of and scoring mechanism in the game.

#### Game Scenario Description and Narrative

The ED receives a predefined number of incoming patients, who are triaged each round based on actual patient flow data from a large pediatric ED. Decisions need to be made by the triage nurse regarding whether the patient is referred or stays in the ED.

**Figure 1:**
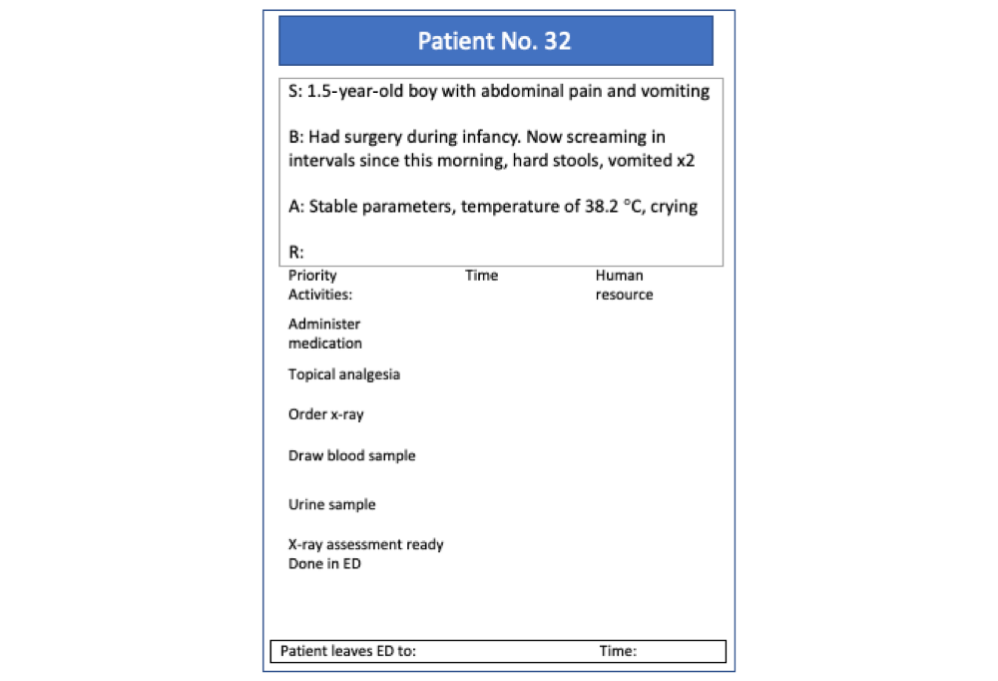
Game card representing a patient. ED: emergency department. SBAR: situation, background, assessment, recommendation.

The serious game is designed to facilitate the practice of core NTSs in a health care production environment. The patients are represented using profiles, as illustrated in Figure 1. There are 90 such unique profiles. Patients are prioritized before being diagnosed or treated by doctors and nurses who work at the modules. Each patient requires an individual resource plan from the responsible person at the appropriate module. The use of stretchers and the priority levels of patients are decided using negotiation between modules and the triage station. Urgent patients, the only level indicated on the profile, must be sent to the red module. The game encourages the organizational dynamic between individual key performance indicators and a high-performance working system at the organizational level with a better patient flow and shorter lead time.

#### Application of Motivational Theory in Serious Gaming for Logistical Aspects

**Figure 2:**
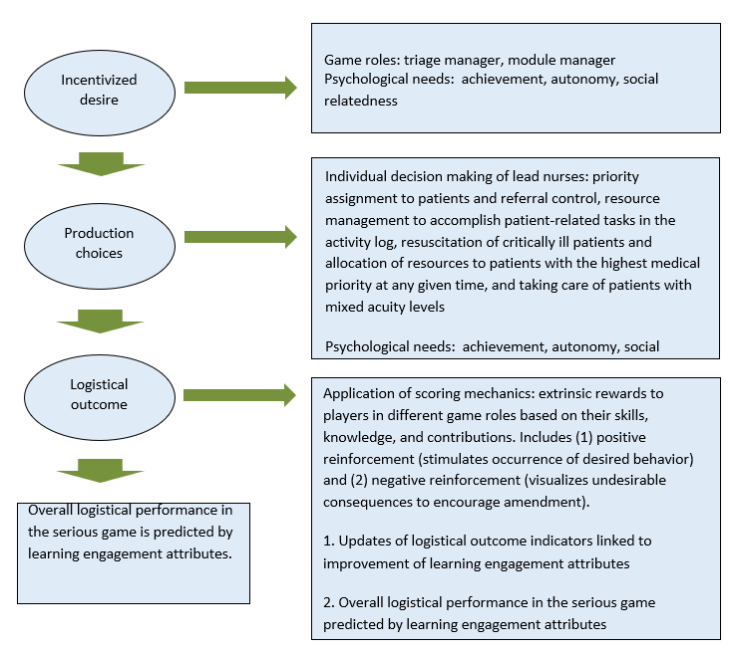
Reinforcement theory of motivation applied to serious gaming for logistics processes in emergency medicine.

As the learning goal of the game was to practice NTSs in the dynamic resource management scenario of a pediatric ED that faces challenges related to case mix and low-acuity inflows, the design focused on encouraging participants to work toward the desired system performance and visualize less effective behavior so that it could be used for reflection and learning. Although previous studies have been based mostly on the fulfillment of players’ psychological needs [34,35], the game design in this study was based on reinforcement theory as a motivational theory that is widely and successfully used for procedural training and education in health care production settings (Figure 2).

The reinforcement theory of motivation focuses on what happens to an individual when he or she takes action to enable a controlling mechanism in line with organizational goals. Both positive and negative reinforcement are integrated with all logistical outcome indicators to offer positive responses when the participant makes the desired choice related to the flow (ie, implicitly this means the throughput time and the quality of the production), leading to a plausible logistical outcome and reducing the possibility of repeating undesirable production choices. The reward system therefore rewards a decision that is in line with the mapped real processes and punishes ones with deviation, which is a typical behavioristic approach that, when applied in a game with immediate feedback, will have a motivational trigger effect. Thus, regarding the measurement of learning engagement, several proxy attributes were used to describe how active participants were in the serious game (Figure 2). First, the number of addressed tasks was measured. The players address tasks by initiating an investigation of patient profiles, allocating resources, and coordinating with other lead nurses. The activity of the player was measured based on how many tasks were initiated and in how many tasks the player was involved. Second, we measured the time spent on the task. The rationale was that this would sufficiently indicate the focus of the player in carrying out their task. Increased time performing operational tasks represents a moderate relationship with achievement. Third, we measured the number of finished tasks. This is a learner-generated metric on the number of tasks correctly answered. The measurements of concentration are useful for understanding participants’ cognitive load in learning and for user behavior modeling analysis. Figure 2 describes the relationship of the 3 measures used for user engagement and motivation to the 3 types of game activities and explains which psychological needs they serve. This impacts the game flow and the cognitive workload and immersiveness the player feels while playing and triggers the training of procedural knowledge.

#### Scoring-Based Mechanic for Logistical Aspects

As described in the introduction, an important factor that supports the learning process while playing is the inbuilt feedback the player receives during gameplay. Consequently, the game mechanic needs to be meaningful for the player (ie, based on the inbuilt feedback). The player needs to understand if the decision or process execution they just performed in the game is correct. As described in the “Game Scenario Description and Narrative” section, the narrative comprises the core of the learning process and focuses on acquiring procedural knowledge. This process is triggered by events. The focus is on improving the flow and resource usage. This needs to be reflected in the feedback and scoring mechanism both to support the player in performing the task while playing and to measure if the player made the right decisions. A possibility of doing this is to use the same metrics in the game as used for measuring logistics performance, which are typically related to time, quality, and costs. A good basis for this is the level 1 metric in the supply chain operations reference model [36], which can be used for both rewarding a desired or correct decision or for penalizing a wrong execution.

The relevant indicators in each process of the gameplay used for scoring the player performance included production throughput, activity executions, resource management, and production resilience. For production throughput, a point is rewarded when a patient is discharged from the emergency department. Activity executions, based on a comparison of an individual resource plan in the gameplay and the reality, are performed for each simulated incoming patient in order to impose penalty points for any delays of production. This scoring mechanism contributes to process quality and on one side shows deviation from the expected procedure and clearly indicates if medical activities as registered in the patient log are carried out in the right modules. For resource management, penalty points are imposed for the absence of the right staff in corresponding activities. The requirements of doctors and nurses refer to the estimation from subject matter experts based on the operational practice. This is also related to process quality and is implemented in order to point out the importance of planning resources (in this case, the medical staff with the right qualifications). Production resilience is represented by the availability of doctors, as they are the core competent medical employee in almost all kinds of medical care. This scoring mechanism is introduced to ensure that the players pay attention to the aspects of flexibility and adaptability to react to unexpected events (eg, getting more patients than planned, a critical person getting sick, or a device not functioning). It is related to the risk appetite an organization needs to have.

These 4 different mechanisms are implemented in the different processes in the gameplay. The implementation and how it is applied in the game is shown in Table 1, which considers the production and resource characteristics of pediatric emergency medicine. Provided that the positive and negative feedback framework is psychologically beneficial for systematically effective employee performances in operational settings, as demonstrated by Losada and Heaphy [37], the optimal ratio of 6:1 for the distribution of positive and negative feedback is used for the calibration of the order of magnitude for each role in emergency medicine delivery.

**Table 1:** Overview of the scoring-based mechanic for corresponding logistical aspects in the pediatric emergency medicine case study.

Indicator	Scoring-based game mechanics		
	Red module responsible	Normal modules responsible	Triage place
Production throughout	+16 tokens	+4 tokens	A quarter of the tokens the receiving module earns but –1 token for every 2 rejections
Activity execution	–4 tokens for +25%; –8 tokens for +50%; –12 tokens for +75%; –16 tokens for +100%	–1 token for +25%; –2 tokens for +50%; –3 tokens for +75%; –4 tokens for +100%	N/A^a^
Resource management	–4 tokens	–1 token	–1 token
Production resilience	–4 tokens	–1 token	N/A

^a^N/A: not applicable.

### Game-Based Learning Practices

The players in the pilot study evaluating the game effect were recruited from master’s students participating in relevant courses from the Department of Biomedical Engineering and Health Systems. A total of 36 students were recruited for the sandbox game and web-based multiplayer serious game in 2019 and 2020, respectively. The participants received a briefing about the roles, resources, and rules of the game.

#### Learning Behavior Analysis Based on In-Game Data

In-game behavior data were collected. As an initial point of activating the behavior chain, the player was informed of any changes to the point system. All logistical aspects were considered. The behavior coding was then performed in accordance with Table 2. All selected behaviors shown in Table 2 are based on previous studies, with necessary adjustments according to the content of the serious game and the research questions in this study. The data were automatically exported from the multiplayer serious game application and manually judged based on tape recordings of the playing of the board game. Then, the raw data set was created by transcribing in-game behaviors into learning engagement attributes. The logistical performance dependent variable was computed as the number of tokens during a participant’s entire training session.

**Table 2:** Participatory behavior coding guidelines.

Behavior (code)	Account
Managing resources (MR)	The participant’s resource management and coordination activities for emergency medicine personnel
Editing profiles (EP)	The participant’s editing of basic patient information, including titles, tags, and classifications
Inviting cooperation (IC)	The participant’s efforts to share with the rest of the team for managing resources or editing profiles
Finishing tasks (FT)	The participant’s confirmation of decision-making results in the simulated system

#### Gamification Acceptance Evaluation Based on Questionnaire

After playing, each player was asked to answer the Core and Social Presence Modules of the Game Experience Questionnaire (GEQ) validated from a previous study to obtain insights into the experience of gamification and gaming with a digital game practice and multiplayer board game [38]. The main goal of the questionnaire was to evaluate whether the game mechanics were accepted and whether the gamified processes were easy to play and understand. The dimensions were competence, sensory and imaginative immersion, flow, tension or annoyance, challenge, negative affect, and positive affect. The answers to these questions were based on a 6-point Likert scale, with 0 meaning “not at all” and 4 meaning “extremely.”

In addition, the participants answered a questionnaire that featured 3 dimensions of social presence based on a 6-point Likert scale, with 0 meaning “not at all” and 4 meaning “extremely.” These dimensions were psychological involvement – empathy, psychological involvement – negative feelings, and behavioral involvement.

#### First Case Study: Sandbox Game

**Figure 3:**
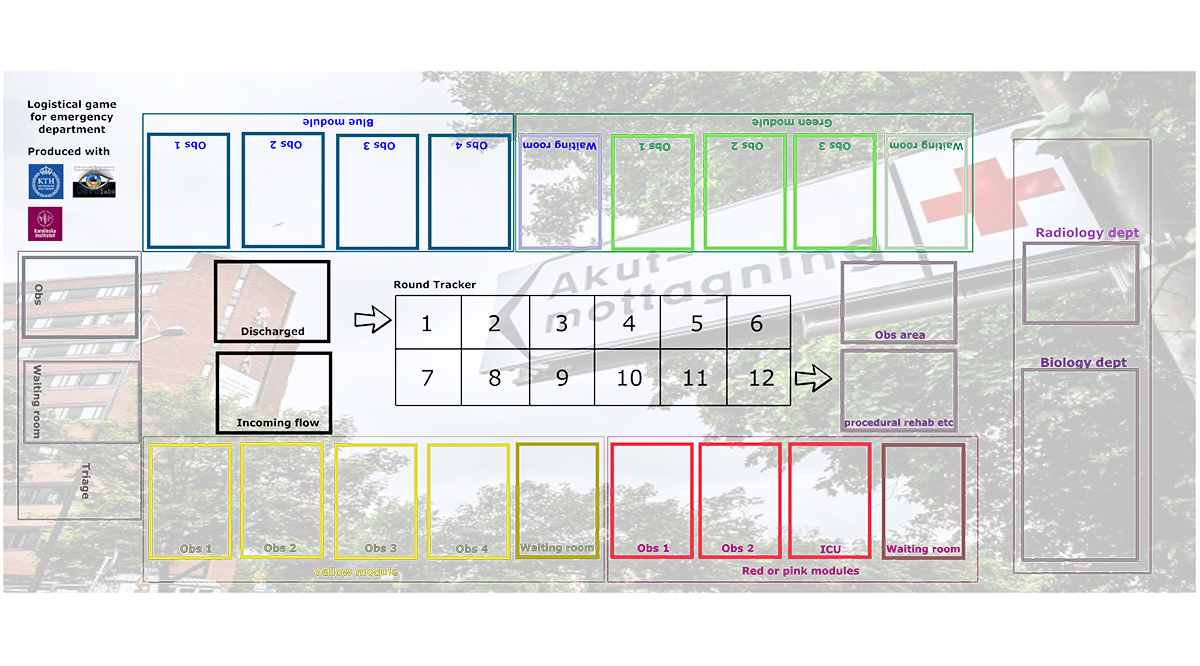
Sandbox game for simulating emergency medicine. Dept: department; obs: observation.

The first case study was based on an analog sandbox game (Figure 3). The game requires participation from 5 module nurses, simulating 5 modules, and 1 individual management nurse, playing the triage place in the ED. The team under the control of the management role is composed of physicians and nurses who take care of all activities.

The ED receives a predefined number of incoming patients for triage each round based on actual patient flow data from a large pediatric ED, and decisions must be made by the triage nurse regarding whether the patient is referred elsewhere or stays at the ED.

The patients who stay at the ED are prioritized and then cared for by the simulated teams of doctors and nurses who work at the workstations or modules. The session lasts 12 rounds, simulating 6 hours in real time. Each patient has a unique profile for prioritization and activities, requiring human resources that are allocated by the person responsible for the module. The observation rooms and patient prioritization are decided by the module nurses and triage nurse, with urgent patients indicated on cards sent to the red module as an exception.

#### Second Case Study: Multiplayer Online Serious Game

**Figure 4:**
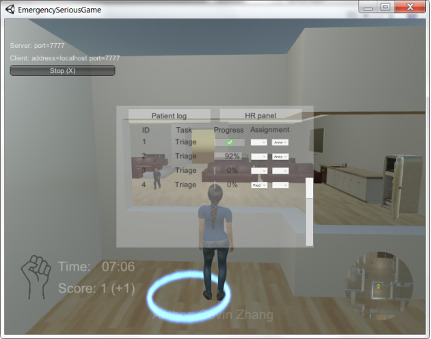
Screenshot of the Unity-based multiplayer serious game. HR: human resources.

The second case study took place via teleconference, with the same participants playing a serious game programmed in Unity (Unity Technologies). An online gaming lobby was available before each session from the built-in UNet service. The players were prompted by the workstation indicators if there were tasks assigned to their specific modules. Resource management occurred through the digital protocol when the players entered the working areas (Figure 4). In addition, the players could navigate the simulated emergency department and interact with nonplayer characters, such as patients, doctors, and nurses. Unlike the sandbox game in the first case study, the multiplayer online serious game automatically calculated and presented the player’s score. It is important to note that the actual game mechanics were identical between the analog and digital game.

### Ethical Approval and Considerations

The study sought ethical approval from Stockholm’s regional board of ethical approval, which resulted in the decision that the use and processing of any data in this study do not require ethical approval (case No. 2019-0744).

Participation in the games did not affect students’ grades and great care was taken to ask for students’ voluntary participation, apart from any mandatory learning activities. The examiner of the courses involved did not attend the gameplay sessions and was not informed about the results to avoid potential cross contamination.

## Results

### Participants

The data set of the serious game consisted of 5263 rows of raw data–containing information. The players’ average age was 25.72 (SD 6.64) years. The students spent a total of 178 hours interacting with the digital environment. The students in the sample were from management and engineering backgrounds.

### Player Questionnaire

**Figure 5:**
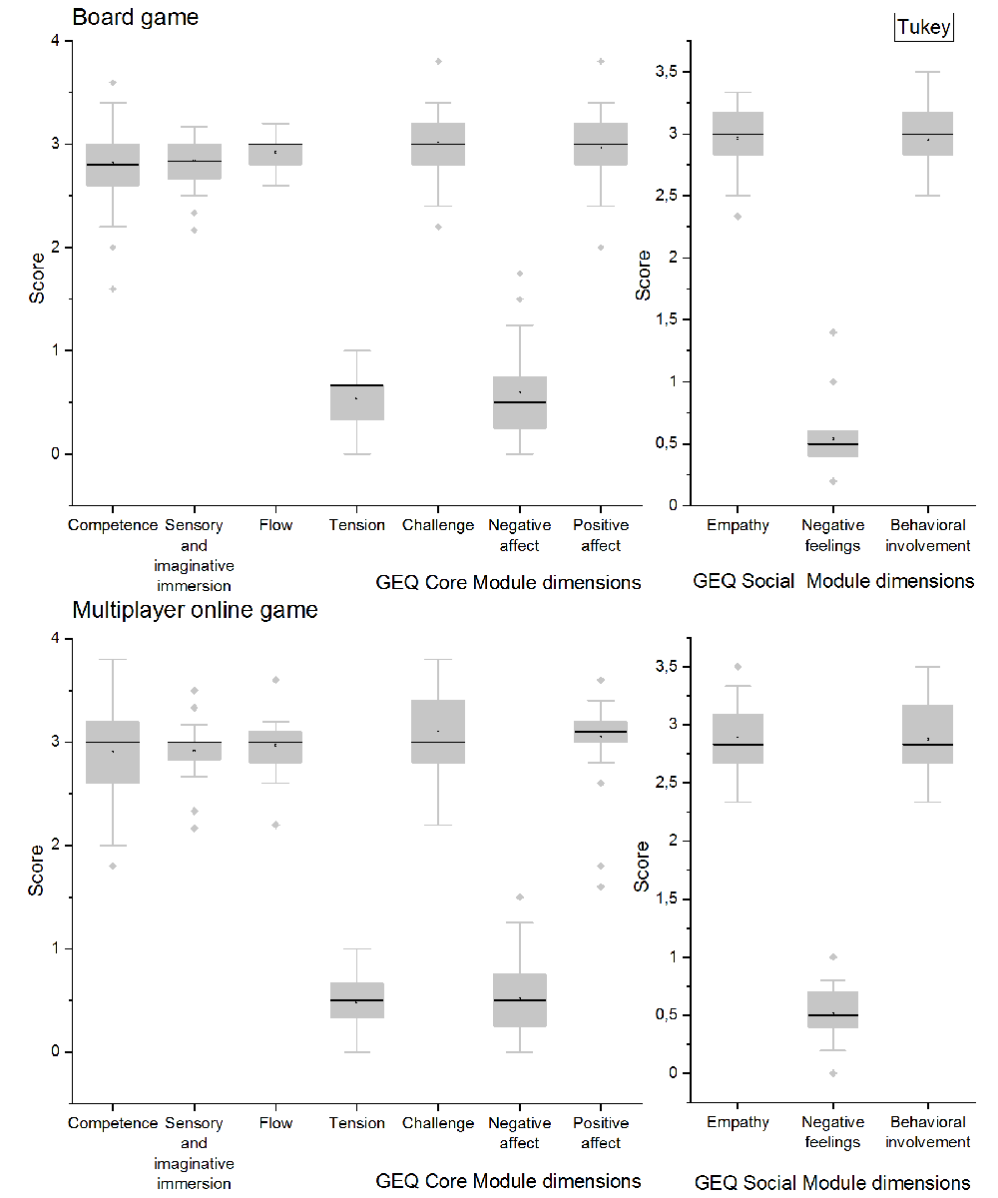
Player questionnaire results for both versions of the game. GEQ: Game Experience Questionnaire.

In the feedback from both cases, all respondents indicated that the game realized in either modality was easy to play and understand, as Figure 5 illustrates. The Core Module items displayed high values for positive affect, challenge, and flow and low values for tension and negative affect. The assessment of the game experience revealed a significant score difference between either tension or negative affect and any of the remaining dimensions. The social presence value reported scores varying between 0.5 and 3 across 3 dimensions. Empathy and behavior involvement scored higher in the board game practice. No component-specific significant difference was reported when the modality of the game was a discriminating factor.

### Lag Sequential Analysis of Learning Behaviors

**Figure 6:**
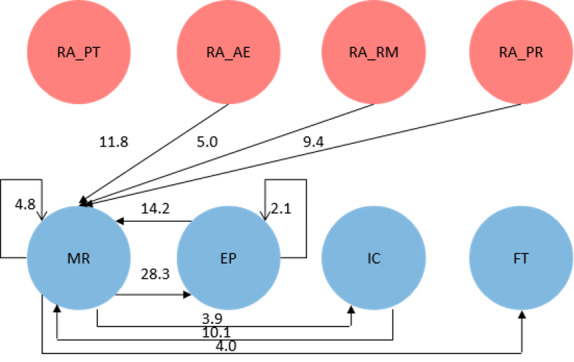
Behavior transition graph for the digital multiplayer serious game. AE: activity executions; EP: edit profiles; FT: finish tasks; IC: invite cooperation; MR: manage resources; PR: production resilience; PT: production throughput; RA: receiving update; RM: resource management.

**Figure 7:**
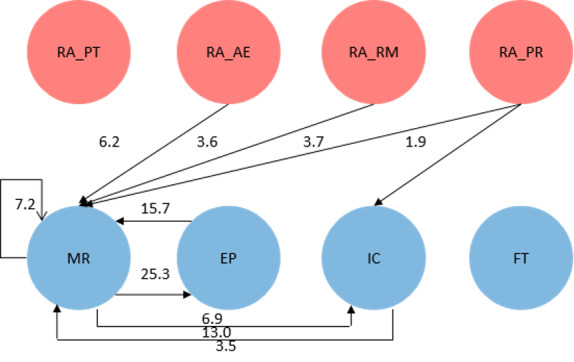
Behavior transition graph for the board game. AE: activity executions; EP: edit profiles; FT: finish tasks; IC: invite cooperation; MR: manage resources; PR: production resilience; PT: production throughput; RA: receiving update; RM: resource management.

During the process of content generation, the participant had access to circulating management resources (manage resources → manage resources: *z* score of 4.8 for the multiplayer serious game and 7.2 for the board game) and could edit profiles (edit profiles → edit profiles: *z* score of 2.1 for the multiplayer serious game only) within the time horizon, as presented in Figures 6 and 7. As long as the participant finished the resource management work, the participant probably proceeded to the work of inviting cooperation (manage resources → invite cooperation: *z* score of 3.9 for the multiplayer serious game and 2.1 for the board game) or editing profiles (manage resources → edit profiles: *z* score of 28.3 for the multiplayer serious game and 25.3 for the board game). Inviting cooperation was preliminarily followed by resource management (invite cooperation → manage resources: *z* score of 10.1 for the multiplayer serious game and 13.0 for the board game). In the learning process, the participant acted after receiving updates from the gamification features of activity execution, resource management, and production resilience. In addition, the scoring-based game mechanics on the logistical aspect of production throughput merely activated higher-order learning behaviors. Players’ synthesizing, reasoning, and evaluation were visible when they actively interacted with artifacts in this simulated environment, delivered learning behavior transitions, and were informed about logistical aspects of the pediatrics ED.

### Learning Engagement and Logistical Performance

The standard regression analysis for the physical board game and the multiplayer online game is presented in Tables 3 and 4, respectively. For the physical board game and the multiplayer online serious game, the output yielded a ΔR^2^ of 0.796 and 0.702, respectively (F1_4,31_=2725.49, *P*<.001; F2_4,31_=2635.31, *P*<.001). Clearly, the number of activities initiated by a player (β=0.42, *P*<.001 for the board game; β=0.34, *P*<.001 for the online game) positively anticipated his or her logistical performance. The same was true for the number of activities finished by a player (β=0.53, *P*=.045 for the board game; β=0.37, *P*=.04 for the online game) and the effort of editing profiles (β=0.08, *P*=.01 for the board game; β=0.14, *P*<.001 for the online game). In sum, the 4 hypotheses were accepted for serious gaming for pediatric emergency medicine, confirming significant relationships between attributes of learning engagement and logistical performance in the game.

**Table 3:** Regression analyses predicting logistical performance based on attributes of learning engagement in the board game case study.

Variables	R^2^	ΔR^2^	B (SE)	β	*P* value
Logistical performance	0.796	0.796	N/A^a^	N/A	N/A
Manage resources	N/A	N/A	1.129 (0.014)	0.42	<.001
Edit profiles	N/A	N/A	0.003 (0.002)	0.08	.01
Invite cooperation	N/A	N/A	0.012 (0.006)	0.18	.02
Finish tasks	N/A	N/A	0.154 (0.089)	0.53	.045

^a^N/A: not applicable.

**Table 4:** Regression analyses predicting logistical performance based on attributes of learning engagement in the multiplayer online serious game case study.

Variables	R^2^	ΔR^2^	B (SE)	β	*P* value
Logistical performance	0.702	0.702	N/A^a^	N/A	N/A
Manage resources	N/A	N/A	1.004 (0.009)	0.34	<.001
Edit profiles	N/A	N/A	0.003 (0.001)	0.14	<.001
Invite cooperation	N/A	N/A	0.025 (0.015)	0.31	.02
Finish tasks	N/A	N/A	0.113 (0.067)	0.37	.04

^a^N/A: not applicable.

### Observations of In-Game Collaboration

Analysis of in-game comments and message broadcasting showed that the participants made more collaborative decisions in later rounds of the game (eg, they would actually handle more challenging tasks at the end of the session). This reflects the increased complexity and visibility of the effects of decisions as the number of patients in the ED increases. In early rounds, the participants were cautious about delaying the patients and attempted to organize flows as efficiently as possible. As more patients entered the ED, the key performance indicators varied among the roles. At the same time, the participants made comments and provided feedback in relation to health care management in the game (eg, the ED was easy to optimize at first, but as the session progressed, the players realized that a teamwork setup was much needed to understand the logistical aspects). This meant that the game facilitated discussions comparing it with real systems, a transition from self-regulated decision making toward collective efforts, and the achievement of learning goals.

In reflecting on the game experience, the players reasoned that without combined decision-making ability, proper management of employees and management of the ED was hard. They also highlighted the managers’ central roles in the coordination of work not only in monitoring but also in facilitating work and encouraging self-reflection and determination.

### Experiences of the Two Game Modalities

**Figure 8:**
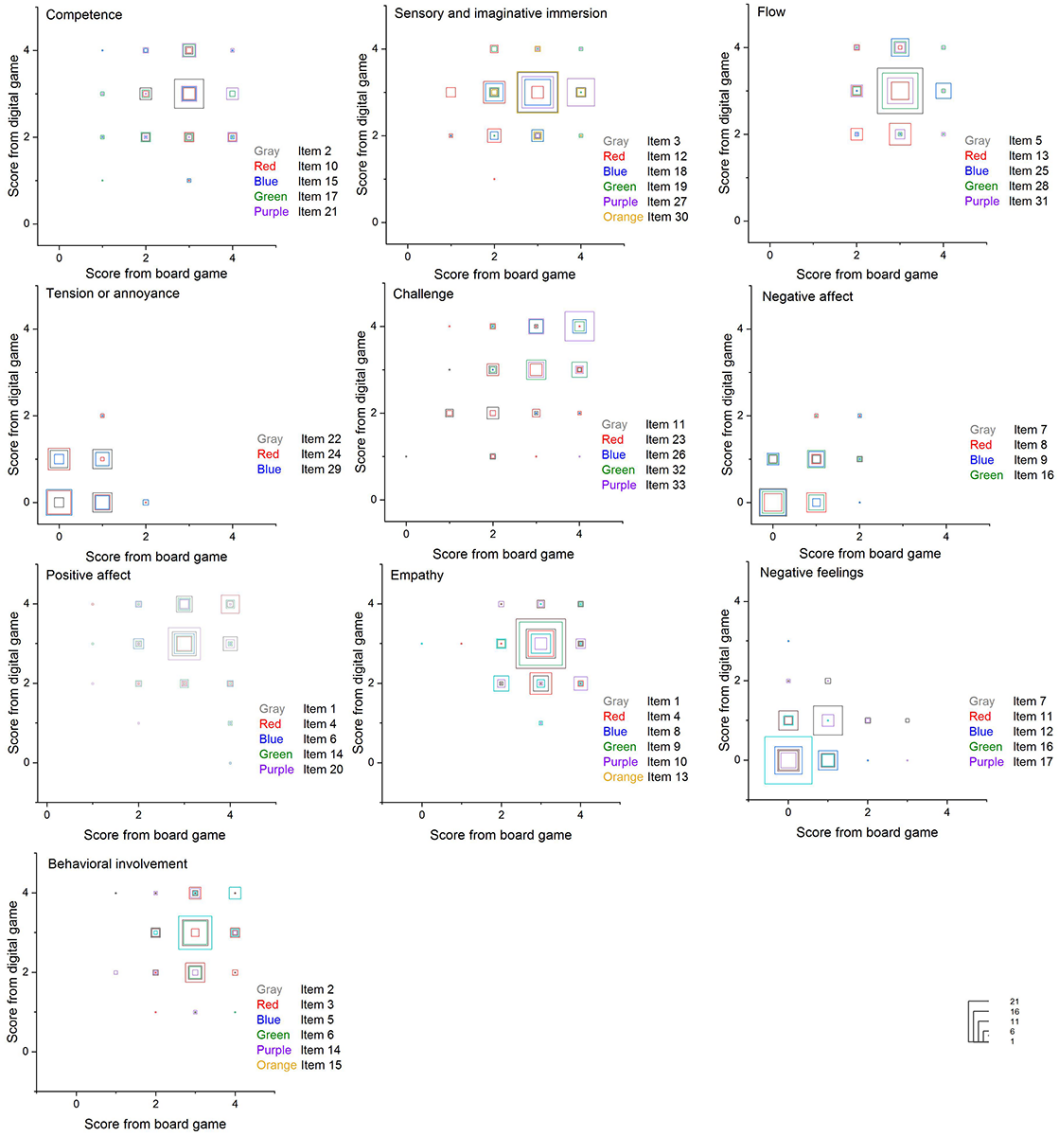
Questionnaire item scores correlated with gaming modalities.

For the challenge dimension, which was scored above 3 for both cases (Figure 8), players in both groups provided mostly positive feedback for item 33 (“I had to put a lot of effort into it”) and item 32 (“I felt time pressure”). However, there were 6 players who indicated slight and moderate confirmations of item 11 (“I thought it was hard”) and item 23 (“I felt pressured”). One potential explanation is that the players in both cases might have been sufficiently challenged by the large number of tasks operated in a limited time horizon in tandem with the narratives, indications, and visual communications of reality, making the game perceived as less difficult. For the negative affect dimension, which was one of the components receiving the lowest scores, players gave mostly slight and moderate confirmation for the questions. The efficacy of the game design is catching score differences tested as statistically insignificant.

## Discussion

### Principal Findings

This study was based on a quantitative investigation of measurable learning engagement attributes and enumerative translation into a serious game of a comprehensive variety of logistical aspects in pediatric emergency medicine. The evaluation was based on the Core and Social Presence Modules of the GEQ to evaluate the experience, feelings, and thoughts related to the serious gaming of logistical processes and management in the health care operation setting.

The results showed that the gameplay of various logistical aspects drives learning engagement attributes, and although the results were weak in strength, they were still significantly positive. Additionally, this study is based on a much larger sample of players participating in both types of gaming simulations. The acceptance and evaluation of the game design corresponded to previous studies on using both physical board simulation and web-based gaming [39], with further improvements.

Provided that both modalities can be applied in training and learning situations [40], the results are remarkable in terms of the impact of the gaming simulation modality on the game mechanics selection; the multiplayer digital version is advantageous for edutainment, whereas the board game version is more straightforward and makes the concepts easy to understand.

This game experience study of the serious game offered meaningful feedback regarding the players’ experiences, which reflects game design principles and realizations. The results indicated an overall positive evaluation for game experience, as most items had values of almost 3 or greater.

Regardless of which particular role is assumed by the players, the survey results demonstrated that the game design was accepted by the players. In addition, the behavior transition graphs from the lag sequential analysis indicated that a point-based system based on mixed positive and negative feedback encouraged the desired actions, and the negative feedback, as an essential part of the framework, was effective.

The competence and challenge components are indicators of the reliable evaluation of the learning engagement attributes that the serious game provided because they measure the subjective participation experience of the player’s skill deployment to its full extent. Both components displayed high values compared with previous applications of serious games addressing health-related issues, which aimed for a higher score in competence [41,42]. The high flow and immersion values suggested that the players felt cognitively involved while playing the game. Meanwhile, the moderate-high value for positive affect and low level of negative affect and tensions indicated the motivating impact of the game. Finally, the high values for empathy and cognitive involvement and the low value for negative feeling, as well as the patterns observed regarding in-game collaboration, indicate the suitability of the game model for teamwork situations.

Our analysis shows that the number of activities addressed and the number of activities finished by a player can be reliable sources for predicting the overall logistical performance in a serious game. This is a reflection of the benefits of active learning with simulation. However, our analysis also showed that better-performing learners spent more time on each particular task. This has not been observed in previous empirical studies. Based on the fact that players revised their plans after being informed by the scoring system, this finding may be interpreted as the players becoming acquainted with situations and the goal of the game.

Anticipatory human resource management was identified as a successful strategy for achieving a sustainable working environment when the organizational resilience was confronted with patient inflow surges during the busiest hours of the busiest day, and this principle was built into the learning goals and scoring system. The findings in this study indicated that both negative and positive reinforcements were effective in encouraging behaviors in line with the learning goals of the game. Our findings show that all included learning attributes were important variables for predicting logistical performance in serious gaming and that the number of initialized tasks was the most powerful explanatory variable, in line with previous studies [31]. Additionally, via relation analysis, this study indicates that a gamification design of key logistical aspects of the health care production system can lead to learning engagement in serious gaming. The results show that the manner in which the players developed solutions and innovations to handle the increasing patient flow during the later rounds corresponded to the expectation of the game principles. The in-game scoring system supported the decision-making process in the desired way. This can be seen as a successful first step in implementing scoring mechanics to support the learning of NTSs needed in an ED. Hospital ED organizations need proactive methods to protect them from disruptive events [10].

### Limitations

A methodological limitation is that the average scores for each dimension in the board game case were not significantly different from those reported in the digital game case, but this can be explained by the difference between the item scores in the same component showing a wear-off effect due to the design of the questionnaire. A more structured and systemic evaluation methodology has been proposed and validated by Carvalho et al [22]. This is a suitable framework, given that a favorable view of game-based training for health care logistics has yet to be shown. This framework will be used to measure the learning curve and the gap between gaming outcomes and observed performance outcomes to further support debriefing and evaluation.

During the game co-design process, emergency medicine shareholders noted that competition is not in line with the culture of health care staff. The emphasis is rather on prioritizing the resources to the patients who are sickest. However, the proposed scoring system aimed not to overemphasize the competing aspects but to provide information to the players on how they managed to adhere to the goals through the simulated scenarios. Also, in this first evaluation of the scoring mechanism, all 36 participants were students. To further investigate how the learning goals work for health care, the game needs to be assessed through the involvement of expert players.

### Conclusions

This study shows that the game in both analog and digital forms can capture and render visible the core logistical challenges of managing an ED.

Previous studies have shown the potential of simulations as a tool for visualizing logistical aspects as well as the effects of an unbalanced use of available resources for health care managers [43].

In addition to the aim of making successful strategies visible to drive expected behaviors, our results showed that the translation of a real emergency department into a serious game and scoring system of logistical aspects also made it possible to explore the roles that communication and collaboration play in achieving the overall goal of balancing efficiency and thoroughness in the ED.

Our study shows the potential of the scoring system and the ability of the game to capture core logistical as well as safety- and quality-related aspects of managing emergency care. A simulation-based game mirroring a real work process, as presented in this study, would be of great value to teach staff tasked with patient flow management about logistical aspects and collaboration in an ED setting.

However, the game has not been tested by expert operators and thus can only demonstrate how players behaved in the context of prompts during gameplay rather than how they would behave based on experiences working in an ED. Therefore, in the next step, the presented results will be used to refine the built-in scoring system and make the tasks more in line with situations in an ED. This will then be tested both with students in next year’s class from the same course and with ED staff.

## Abbreviations

ED: emergency department
GEQ: Game Experience Questionnaire
NTS: nontechnical skill
